# LKD-YOLOv8: A Lightweight Knowledge Distillation-Based Method for Infrared Object Detection

**DOI:** 10.3390/s25134054

**Published:** 2025-06-29

**Authors:** Xiancheng Cao, Yueli Hu, Haikun Zhang

**Affiliations:** 1 School of Mechatronic Engineering and Automation, Shanghai University, Shanghai 200444, China; caoxiancheng@shu.edu.cn; 2 School of Mathematics-Physics and Finance, Anhui Polytechnic University, Wuhu 241000, China; haikunzhang@ahpu.edu.cn

**Keywords:** infrared object detection, knowledge distillation, attention mechanism, edge computation

## Abstract

Currently, infrared object detection is utilized in a broad spectrum of fields, including military applications, security, and aerospace. Nonetheless, the limited computational power of edge devices presents a considerable challenge in achieving an optimal balance between accuracy and computational efficiency in infrared object detection. In order to enhance the accuracy of infrared target detection and strengthen the implementation of robust models on edge platforms for rapid real-time inference, this paper presents LKD-YOLOv8, an innovative infrared object detection method that integrates YOLOv8 architecture with masked generative distillation (MGD), further augmented by the lightweight convolution design and attention mechanism for improved feature adaptability. Linear deformable convolution (LDConv) strengthens spatial feature extraction by dynamically adjusting kernel offsets, while coordinate attention (CA) refines feature alignment through channel-wise interaction. We employ a large-scale model (YOLOv8s) as the teacher to imparts knowledge and supervise the training of a compact student model (YOLOv8n). Experiments show that LKD-YOLOv8 achieves a 1.18% mAP@0.5:0.95 improvement over baseline methods while reducing the parameter size by 7.9%. Our approach effectively balances accuracy and efficiency, rendering it applicable for resource-constrained edge devices in infrared scenarios.

## 1. Introduction

Machine vision [1,2], as a vital component of artificial intelligence, has undergone rapid and substantial advancement over the past twenty years. With the continuous advancement of computer vision technology. Infrared target detection has become increasingly widespread in fields such as military, aviation, and healthcare. Infrared target detection plays a significant role within the domain of computer vision, which involves detecting objects in infrared images using infrared thermal imaging technology. Although optical cameras used in daily life can flexibly capture high-resolution images from optical sensors, their imaging performance deteriorates sharply in complex natural environments, such as at night or in the presence of smoke, fog, or dust, making object recognition extremely challenging. In these conditions, infrared detectors provide more stable imaging, making thermal cameras capable of detecting objects based on the thermal radiation emitted within the long-wavelength infrared (LWIR) spectrum. However, the typically low resolution of commercial thermal cameras results in infrared images that exhibit limited detail and clarity, thereby impeding the efficacy of target detection. Hence, achieving high-precision detection of infrared targets is of critical importance.

Deep learning techniques have recently emerged as indispensable tools in the field of infrared object detection. For example, The R-CNN [3,4,5] series of networks and the YOLO (You Only Look Once) [6,7,8,9] series of single-stage networks have both achieved superior performance over traditional methods in infrared object detection tasks. However, it should be emphasized that the algorithms utilized in infrared detection scenarios predominantly rely on edge devices, which aim to achieve efficient object detection techniques that meet real-time processing requirements. While YOLO outperforms earlier detection models, deploying it on edge devices remains arduous due to their limited computational resources. Consequently, a critical issue that requires immediate attention is the development of a lightweight implementation of the YOLOv8 model, while ensuring the preservation of its high performance.

The YOLOv8 algorithm, renowned for its advanced object detection capabilities, offers multiple advantages, including real-time performance, architectural simplicity, computational efficiency, multi-scale detection and integration of global contextual information. These features collectively enhance its effectiveness in rapid object detection and real-time applications. However, despite these strengths, the YOLO series has historically encountered challenges related to excessive parameterization and substantial computational demands, particularly when computational resources are limited. Consequently, the development of lightweight versions of YOLO models that maintain robust object detection performance has become a critical focus area for researchers. Endeavors in this direction aim to optimize the compromise between model precision and inference efficiency, facilitating the deployment of YOLO models in resource-constrained environments.

To address the aforementioned issues, researchers have proposed abundant model acceleration and compression techniques. These include quantization, pruning, lightweight model design, and knowledge distillation, all of which aim at minimizing model deployment requirements and accelerating inference by reducing the numerical precision of weights and activations. Knowledge distillation is a prominently used model compression method in which a compact “student” network is guided to replicate the predictive behavior of a larger, high-capacity “teacher” model. This approach enables the student model to inherit the performance advantages of the teacher while significantly reducing computational overhead. In contrast to methods such as pruning and quantization, knowledge distillation entails the development of a compact model, which is subsequently trained utilizing the supervisory information derived from a more proficient, larger model. This approach aims to achieve better performance and accuracy, which is initially proposed and applied by Hinton [10] in 2015 for classification tasks. The larger model is commonly termed the “teacher,” whereas the smaller, compressed model is known as the “student.” The informative guidance extracted from the teacher’s output is regarded as “knowledge,” and the process through which the student acquires this knowledge is known as “distillation”.

Despite the remarkable progress in infrared object detection, a critical research gap remains in developing models that can simultaneously ensure high detection accuracy while meeting the stringent computational constraints of edge devices. Existing lightweight detectors often suffer from degraded performance due to their limited representational capacity, whereas more accurate models are typically too resource-intensive for real-time deployment. Although knowledge distillation has shown considerable promise in compressing models without significantly sacrificing accuracy, most previous studies have primarily focused on logit-based or feature-level distillation approaches, which often lack sufficient semantic and spatial supervision in infrared-specific contexts. The unique characteristics of infrared imagery—such as low contrast, small object sizes, and high noise levels—demand more effective distillation strategies tailored to these challenges. However, limited attention has been given to integrating advanced distillation techniques with architectural enhancements specifically designed for infrared detection. This study aims to bridge this gap by proposing a distillation-aware lightweight YOLOv8 framework that leverages knowledge distillation adaptive convolutional modules and attention mechanisms to achieve efficient and accurate infrared object detection.

Inspired by knowledge distillation [11,12,13,14,15], we propose a lightweight knowledge distillation-based YOLOv8 network (LKD-YOLOv8), a novel infrared object detection method that integrates the YOLOv8 architecture with the masked generative distillation (MGD) method [16]. In LKD-YOLOv8, the YOLOv8s model functions as the teacher network, supplying soft labels that assists the training process of the YOLOv8n model. To enhance spatial feature adaptability and reduce the computational cost of convolution, we incorporate LDConv [17], which dynamically adjusts kernel offsets. Infrared objects typically appear as small targets with varying characteristics due to environmental conditions, necessitating effective multi-scale feature extraction. Therefore, CA [18] is utilized to refine feature alignment through channel-wise interaction, thus improving the detection accuracy of small targets by enhancing the model’s ability to focus on fine-grained spatial details. Our approach enhances both localization precision and contextual information utilization while maintaining computational efficiency. The primary contributions of this study are delineated as follows:

- We propose an innovative framework for infrared object detection, LKD-YOLOv8, which leverages knowledge distillation to improve detection accuracy while maintaining a lightweight network suitable for real-time processing.
- LKD-YOLOv8 incorporates LDConv and CA. LDConv enhances spatial feature extraction by dynamically adjusting kernel offsets. CA improves feature alignment through channel-wise interaction, thereby enhancing the perception ability of small or blurred infrared targets.
- By distilling a lightweight student model (YOLOv8n) with a larger teacher model (YOLOv8s), our method effectively transfers knowledge and achieves a balance between model effectiveness and resource expense. The experimental results demonstrate that LKD-YOLOv8 outperforms baseline methods, improving mAP@0.5:0.95 by 1.18% while reducing parameter size by 7.9%.

## 2. Related Work

### 2.1. Model Compression and Acceleration

Deep learning technologies have greatly accelerated advancements in fields such as machine vision and text translation, primarily by leveraging large-scale pre-trained models to boost the effectiveness of deep neural networks across a wide range of tasks. However, the substantial parameter complexity and computational demands of these models pose significant challenges for deployment on mobile devices, thereby necessitating the development of model compression and acceleration techniques. Mainstream methodologies include model pruning [19,20,21,22], knowledge distillation, and tensor decomposition [23,24,25], which can be specifically described as:

- Model pruning, which simplifies the model by discarding redundant weights and network links, thereby streamlining the model architecture without compromising performance.
- Knowledge distillation, which leverages a high-capacity teacher model to guide the training of a more compact student model, effectively transferring knowledge and facilitating the improvement of lightweight models.
- Tensor decomposition, which decomposes tensors to reduce the dimensionality of the model, thus decreasing storage requirements and enhancing computational efficiency.

Knowledge distillation techniques can be roughly categorized into two primary approaches: feature-based distillation methods [26,27,28,29,30], which focus on transferring intermediate representations and logit-based distillation methods [31,32,33,34]. Logit-based knowledge **conventionally** pertains to the response generated by a neuron or the output produced by the final logical layer of the teacher model. Soft targets derived from the teacher’s logits are utilized to inform and direct the student model’s optimization. As a rule, neural networks utilize a softmax activation function in the output layer to convert raw class scores (logits), $z_{i}$, into a probability distribution, $q_{i}$, enabling the interpretation of each class’s likelihood. This transformation is mathematically represented as

(1) $$q_{i}=\frac{\exp(z_{i}/T)}{\sum_{j}\exp(z_{j}/T)},$$

where temperature T is a key hyperparameter that adjusts the “softness” of the probability distribution output by the teacher model. The compact student models are guided by the cross-entropy loss of soft labels to inherit the teacher’s intricate decision boundaries while maintaining computational efficiency.

The knowledge learned by the network is hierarchical, with increasingly higher levels of abstraction from shallow to deep layers corresponding to the knowledge. Therefore, the features in the intermediate layers can also be used to transfer significant knowledge. Feature-based distillation utilizes the features of the middle layer as a carrier of knowledge. A standard mathematical formulation of the distillation loss in feature-based methods is given by [35]

(2) $$Loss_{\mathrm{Feature}}\left(F_{t}(x),F_{s}(x)\right)=Align\left(\partial_{t}(F_{t}(x)),\partial_{s}(F_{s}(x))\right),$$

where $F_{t}(x)$ refers to the teacher’s feature map and $F_{s}(x)$ corresponds to that from the student model, both taken from their respective intermediate layers. To address mismatched feature dimensions, transformation functions $\partial_{t}()$ and $\partial_{s}()$ are applied, while the function Align() operates as a similarity measure to ensure effective alignment of the feature maps.

Finally, knowledge distillation has been proved in enhancing smaller models through supervision from larger models. It exhibits high adaptability to the constraints associated with latency-sensitive detection scenarios.

### 2.2. Attention Mechanisms

To augment feature representation, deep learning models always incorporates attention mechanism. Channel-wise (CHA) and spatial-wise (SA) attention mechanisms are among the most prevalent attention methods, and many studies have demonstrated their effectiveness in enhancing computer vision tasks. To elaborate, CHA operates on the channel dimension, while SA functions on the spatial dimension.

Channel attention: CHA enables neural networks to identify what to focus on. Within deep neural networks, different channels across feature maps encode representations of different objects [36,37]. The weight of each channel is flexibly recalibrated by CHA. This can be regarded as a process of object selection, thereby determining the focal points for attention.

Spatial attention: the spatial attention mechanism [38,39] enables neural networks to find the locations or pixels that should be focused on. By transforming the spatial characteristics of the input image into a new representation space, the SA attention mechanism can reliably retain the critical features. In brief, spatial attention acts as an adaptable spatial region selection mechanism, showing where to pay attention.

By integrating attention mechanisms, infrared object detection models become more adept at focusing on the most relevant features and regions, eventually leading to enhanced detection accuracy and robustness in challenging infrared imaging conditions.

## 3. Proposed Method

**Figure 1:**
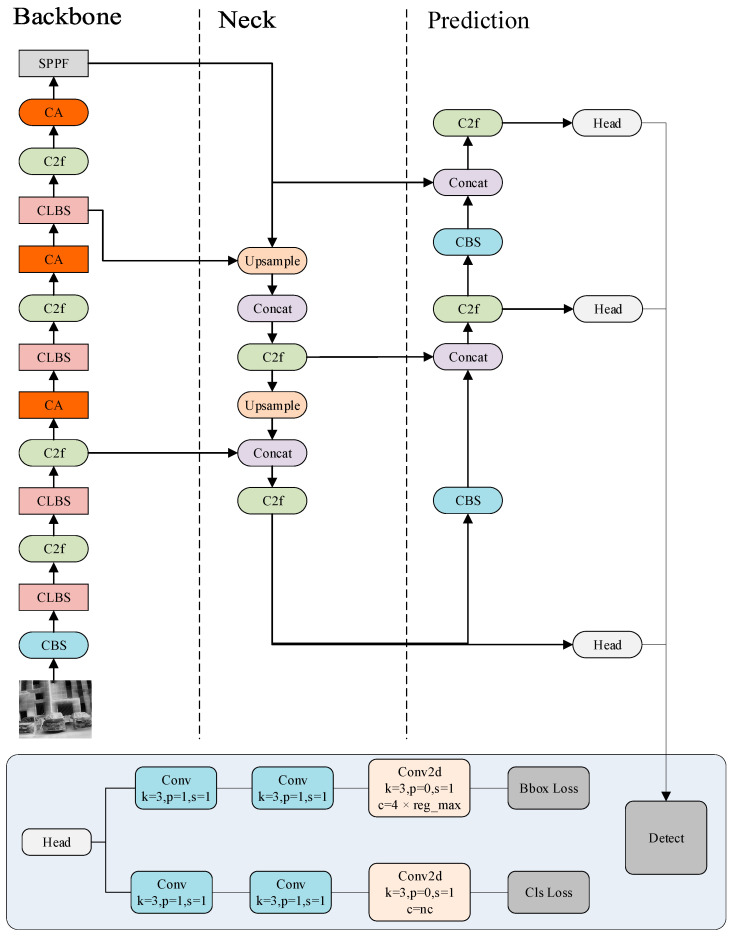
Network structure of LKD-YOLOv8.

**Figure 2:**
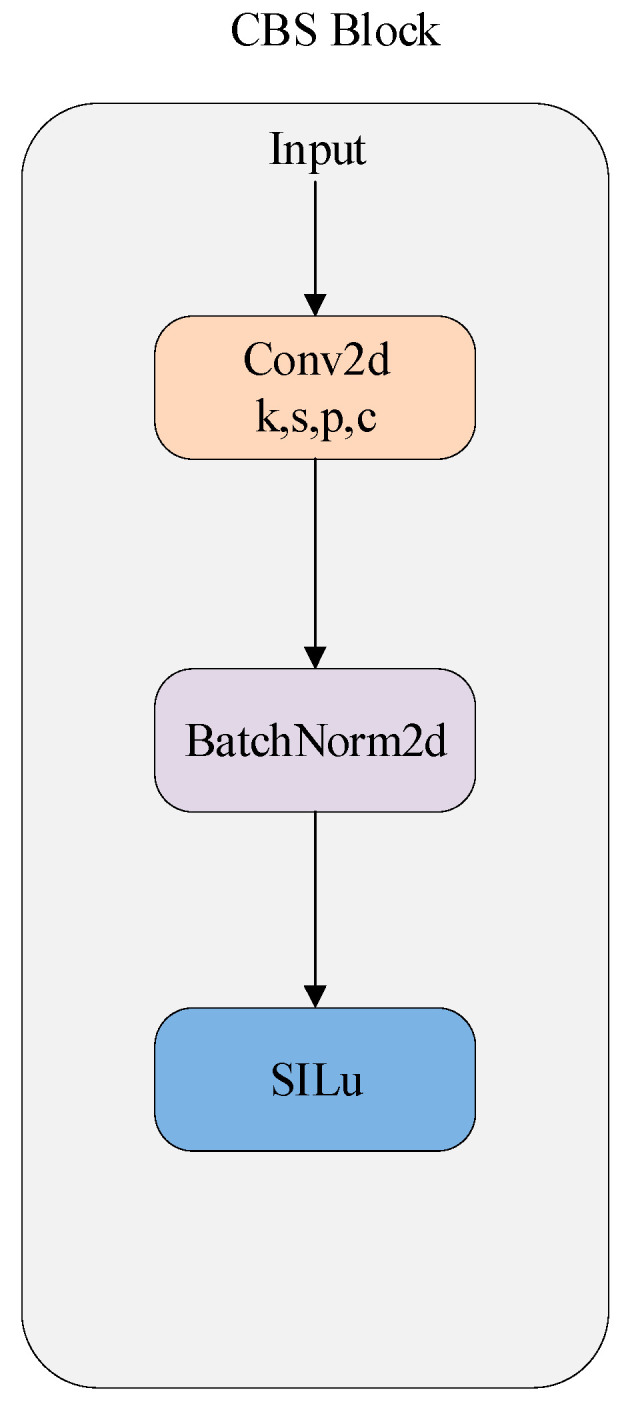
Structure of CBS block. K, s, p, and c stand for kernels, stride, padding and channels, respectively.

**Figure 3:**
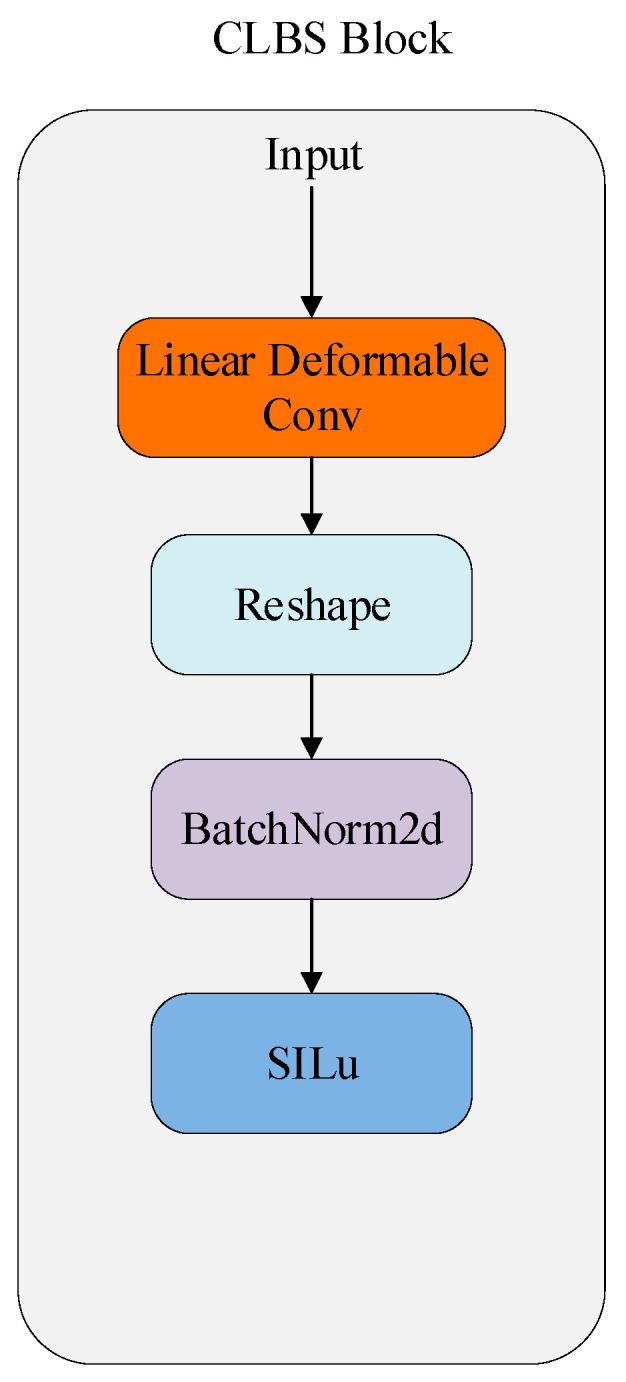
Structure of CLBS block.

The overall architecture is depicted in Figure 1, while the detailed configurations of the CBS and CLBS modules are illustrated in Figure 2 and Figure 3, respectively. As shown in Figure 2 and Figure 3, the key distinction between CBS and CLBS lies in the replacement of the standard convolutional module with a linear deformable convolution in CLBS.

### 3.1. Network Design

The student model shares an identical network architecture with the teacher model, with variations only in parameter numbers and model size. The network structure of LKD-YOLOv8 is modified and optimized based on the origin YOLOv8 model, including the backbone, neck, and detection head. The network model adopts Cross Stage Partial Darknet (CSP) [40] as the backbone, incorporating the CLBS (Convolution + LDConv + Batch Normalization + SiLU) module and the C2f (Cross Stage Partial and 2-Fold Aggregation) module. The CLBS module is an optimized module that substitutes the original CBS (Convolution + Batch Normalization + SiLU) module in the YOLOv8 network. Specifically, in the CBS module, the input first passes through a standard convolution block. In the CLBS module, the conventional convolution block is substituted with LDConv, enhancing feature extraction abilities and adaptability, while simultaneously lowering computational costs and reducing the parameter count. Additionally, after each C2F module, a CA module is incorporated to boost performance with minimal computational overhead.

### 3.2. Masked Generative Distillation

**Figure 4:**
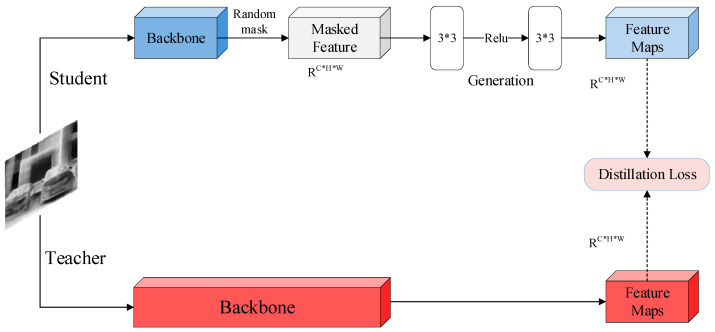
Masked generative distillation procedure.

Beyond feature imitation, the objective of the MGD strategy is to drive the student to actively produce the features. It helps the student in obtaining a deeper understanding of the input feature map. According to the network structure shown in Figure 1, we adopt LKD-YOLOv8-s (small) as the teacher, which is typically well-trained before being employed in the knowledge distillation process. The distillation process is depicted in Figure 4:

The teacher model is supposed to be input before the training process begins. Applying to MGD, a random mask of shape 1×H×W (H and W represents the height and width of the mask, respectively) is generated for the feature map of the detection head module of the student model. This mask is then broadcast across all channels for masking operations. Subsequently, a generation module is employed to generate the complete feature map of the teacher model by using the masked feature map. Throughout each training iteration, random pixels are selected to ensure that every pixel is utilized in the training, thereby strengthening feature reliability and improving representational capacity. Finally, the output feature is compared with the teacher’s feature map to calculate the mean squared error (MSE) loss, and the network parameters are updated through backpropagation. The entire process is carried out repeatedly until the maximum iteration limit is achieved, upon which the student model training is terminated.

Let $Teac^{l}\in R^{C\times H\times W}$ and $Stud^{l}\in R^{C\times H\times W}(l=1,\ldots ,L)$ represent the feature maps from the detection head (C is channel number in the feature map). The application of the l-th random mask to obscure the l-th feature of the student can be expressed as

(3) $$Mask_{i,j}^{l}=\left\{\begin{array}{ll} 0, & \mathrm{if}\;R_{i,j}^{l}<\alpha \\ 1, & else \end{array}\right.$$

where $R_{i,j}^{l}$ denotes a randomly selected value within a specific range. i and j, respectively, correspond to the width-wise and height-wise directions of the feature map. α is a hyperparameter that indicates the proportion of the feature map to be masked. The corresponding mask is then applied to cover the student’s feature map, and the remaining pixels will be used by the student to reconstruct the teacher’s feature map, which is given by

(4) $$P(F_{\left(S^{l}\right)}\times Mask^{l}\to Teac^{l})$$

(5) $$P(F)=W_{l2}(ReLU(W_{l1}(F)))$$

The projector layer P consists of two convolutional layers $W_{l1}$, $W_{l2}$ and one ReLU activation layer. F denotes the adaptive layer that coincides the student features with the teacher features. The distillation loss is

(6) $$S_{dis}\left(Stud,Teac\right)=\sum_{l=1}^{L}\sum_{k=1}^{C}\sum_{i=1}^{H}\sum_{j=1}^{W}(T_{k,i,j}^{l}-P(F\left(S_{k,i,j}^{l}\right)\times Mask_{i,j}^{l}){)}^{2}$$

where S is the sum of layers used in distillation. In general, the overall loss of the training process is

(7) $$L_{all}=\beta \times S_{dis}+L_{origin}$$

where $L_{origin}$ is the loss of the origin model. β serves as a hyperparameter to balance the losses. We set α to 0.67 and β to 0.000002.

### 3.3. Coordinate Attention

Recently, deep neural networks often incorporate attention mechanisms to specify which features and regions the model should focus on. These mechanisms have been extensively studied and applied to boost the performance of neural networks [38,41,42,43]. Because of the limited computation capacity of networks in edge devices, the Squeeze and Excitation (SE) [42] attention are the most widely used attention mechanism. It employs channel-wise attention through the global pooling, resulting in notable enhancements in performance while maintaining a relatively low computational expense. Nevertheless, the Squeeze and Excitation (SE) attention mechanism emphasize on inter-channel information, thereby neglecting spatial cues, which is critical for the accurate identification of target objects.

**Figure 5:**
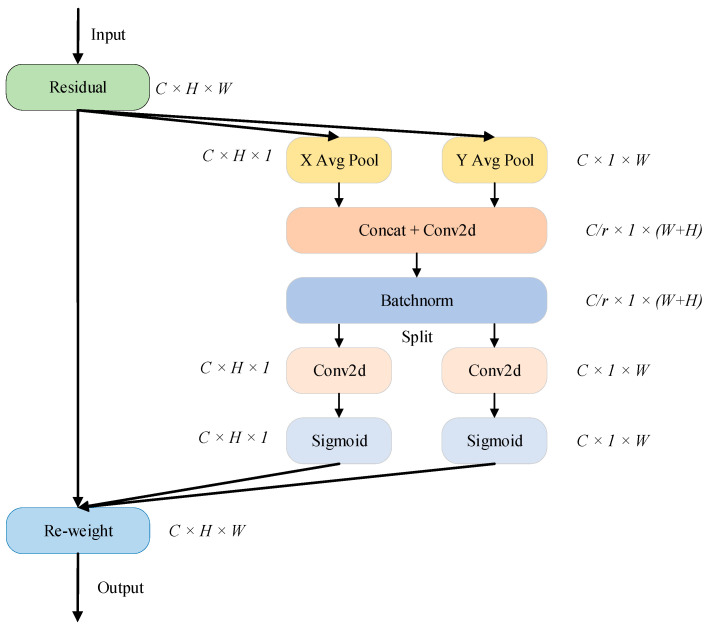
CA’s schematic structure.

The coordinate attention (CA) mechanism employs a bidirectional one-dimensional global pooling strategy to assemble input features along both the height-wise and width-wise axes. This operation produces a dual-channel feature representation that is sensitive to spatial orientation. This mechanism varies from the channel attention (CHA) mechanism, which compresses the input features into a single channel vector through two-dimensional pooling. Specifically, CA encodes and applies nonlinear transformations to features along each spatial axis separately, generating attention weight maps corresponding to the vertical and horizontal dimensions. This axis-specific encoding enables the attention maps to capture distant spatial correlation along each direction and simultaneously retains spatial positional information in the attention weights. As a result, this approach considerably enhances the model’s capacity to distinguish and emphasize salient features. The schematic structure of CA is depicted in Figure 5.

Given the input $x\in {\mathbb{R}}^{c\times h\times w}$, a pooling operation with kernel size (H,1) is applied along the horizontal axis for each channel, which is subsequently followed by vertical pooling using a kernel of size (1,W). The resulting output of the pooling operations are given by

(8) $$d_{c}^{h}(h)=\frac{1}{W}\sum_{0\leq i<W}x_{c}(h,i)$$

(9) $$d_{c}^{w}(w)=\frac{1}{H}\sum_{0\leq j<H}x_{c}(j,w)$$

Afterwards, the feature maps are combined via concatenation and refined using a convolutional function, represented as

(10) $$f=\delta (F(Concat(d^{h},d^{w})))$$

where Concat denotes the concatenation operation, F denotes the convolution transformation, and δ is the activation function. f is an intermediate feature map with dimensions $f\in {\mathbb{R}}^{C/r\times (H+W)}$, and r is the scaling factor, which is set to 32. Next, along the spatial direction, the feature map is divided into two separate tensors: $f^{h}\in {\mathbb{R}}^{C/r\times H}$ and $f^{w}\in {\mathbb{R}}^{C/r\times W}$.

Two 1×1 convolution transformations $F_{h}$ and $F_{w}$ are then applied to convert $f^{h}$ and $f^{w}$ into tensors $g^{h}$ and $g^{w}$, which have the same channels:

(11) $$g^{h}=\sigma (F_{h}(f^{h}))$$

(12) $$g^{w}=\sigma (F_{w}(f^{w}))$$

where σ is the sigmoid function. Both $g^{h}$ and $g^{w}$ function as attention weight maps. Accordingly, the resulting output of the CA module is formulated as

(13) $$y_{c}(i,j)=x_{c}(i,j)\times g_{c}^{h}(i)\times g_{c}^{w}(j)$$

### 3.4. Linear Deformable Convolution

Standard convolution has two main drawbacks. First, it is restricted to local receptive fields, preventing them from capturing information from distant regions. Second, the kernel size is fixed in a square shape, which causes the number of parameters to grow quadratically as the kernel size grows. Although deformable convolution (DCN) [44,45] addresses the issue of fixed sampling positions in standard convolution, its parameter still follows a quadratic growth pattern.

**Figure 6:**
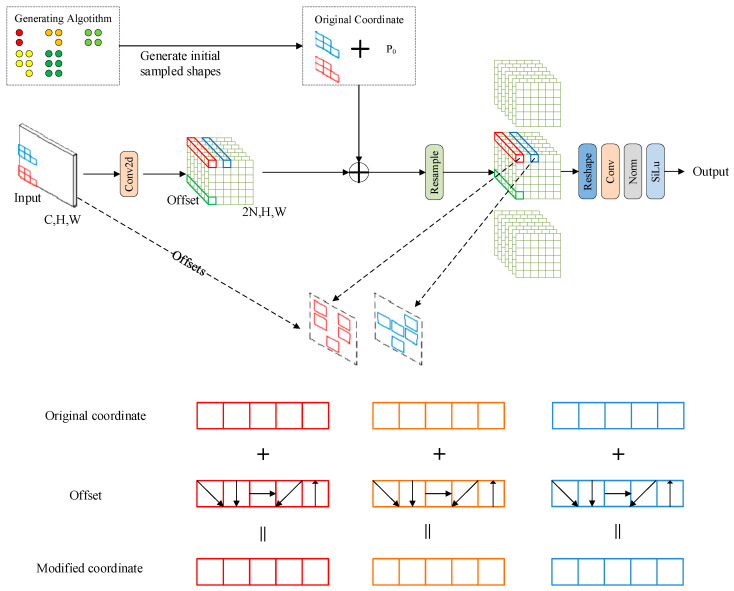
A detailed schematic of the structure of LDConv.

To mitigate these limitations, we incorporate LDConv in place of certain convolutional operations within the original model architecture. The structural design of LDConv is illustrated in Figure 6. LDConv features a flexible kernel design, allowing for arbitrary parameter counts and customizable sampling patterns., offering a broader range of trade-offs between network complexity and performance. With appropriate parameter selection, LDConv is capable of lowering the overall number of network parameters without compromising efficiency. In LDConv, a novel coordinate generation algorithm is proposed to determine initial sampling positions, enabling support for convolution kernels of arbitrary sizes. To adapt to diverse targets, an offset is introduced at each position to adjust the sampling shape. This effectively transforms the quadratic parameter growth trend observed in standard and deformable convolutions into a linear growth pattern, thereby reducing the computational cost of convolution operations.

A coordinate generation algorithm specifies the starting position for convolution kernels of varying sizes, while offsets are applied at each sampling point to handle target variability and dynamically reshape the sampling configuration. This helps to modify the quadratic parameter growth in both standard and deformable convolutions, transforming it into a linear growth trend. LDConv offers more flexibility than deformable convolution and can mimic deformable convolution when its parameters are set to the square of K (kernel size k, *k* = 1, 2…, *K*).

## 4. Experimental Analysis

### 4.1. Dataset

We employ the FLIR Thermal Dataset to train and validate the proposed algorithm, in order to assess its performance in both accuracy and computational expense in infrared object detection. The annotation format does not meet the requirements of our model, so we convert the annotation format to the YOLO format. Furthermore, classes with fewer than 100 instances, which are detrimental to the training process, are excluded from the dataset. In our experiments, we consider seven object classes: person, bike, car, motor, bus, light, and sign. A total of 10,742 images are used for training, and 1144 images are allocated for validation.

### 4.2. Experimental Settings

Our server’s hardware configuration is an Ubuntu 20.04.5 operating system, NVIDIA A100 80GB GPU, Intel(R) Xeon(R) Gold 6254 CPU @ 3.10GHz, 1T GB RAM. The software configuration of our server is Python 3.8.13, GPU driver version 550.90.07, CUDA12.4 and torch 1.13.1; the image size is set to 640 × 640; and the batch size is set to 16 for the teacher model and 8 for the student model. For training the teacher model, the initial learning rate lr0 = 0.01 and lrf = 0.01. For distilling the student model, the initial learning rate lr0 = 0.015 and Irf = 0.015. The SGD optimizer is employed with a momentum value of 0.9 and a weight decay coefficient of 0.0005. The training epochs is set to 1000 with a patience 100.

### 4.3. Evaluation Metrics

The main evaluation metrics used in object detection tasks includes precision (P), recall (R), average precision (AP), and mean average precision (mAP) [46,47]. The mathematical formula is listed below:

(14) $$P=\frac{TP}{TP+FP}$$

(15) $$R=\frac{TP}{TP+FN}$$

(16) $$AP=\int_{0}^{1}P(R)dR$$

(17) $$mAP=\frac{\sum_{i=1}^{N}AP_{i}}{N}$$

where TP represents the instances accurately identified by the model and FP indicates that the model identifies instances that do not exist. FN denotes the ground truth instances that are not successfully detected by the model.

Intersection over Union (IoU) [48,49] quantifies how much the predicted and ground truth boxes intersect, calculated as the overlapping area divided by the total area covered by both boxes. mAP@0.5 means that the evaluation is conducted at an IoU threshold of 0.5, while mAP@0.5:0.95 extends the definition of mAP@0.5 by evaluating the model performance across IoU thresholds ranging from 0.5 to 0.95, in increments of 0.05.

### 4.4. Ablation Experiments

To demonstrate the superior performance of the modules incorporated in the LKD-YOLOv8 network, four ablation experiments are conducted. The corresponding results are summarized in Table 1.

The experimental results in Table 1 indicate the extent to which each module contributes to the overall performance of the model. Group 1 represents the baseline model, which corresponds to the original YOLOv8n model. Group 1 and Group 2 demonstrates the effectiveness of LDConv in lightweight model design. The number of parameters is reduced by 12%, FLOPs decreases by 8.4%, while mAP@0.5:0.95 only decreases 0.08%. By comparing Group 1 and Group 3, we can see that mAP@0.5:0.95 increased by 1.44%, but only introduced extra 0.01 million parameters, which demonstrates the performance improvement brought by CA; therefore, Groups 1 and 4 validate the feasibility of LDConv and CA. When these two modules are jointly functioning, the model’s parameters are reduced by 7.6%, FLOPs decreases by 6.5%, and mAP@0.5:0.95 improves by 0.68%. The reduction in model parameters when combining LDConv and CA compared to using CA alone is primarily attributed to the inherent lightweight nature of LDConv. While CA introduces a small number of additional parameters due to its attention mapping, LDConv significantly reduces parameter counts by replacing standard convolutions with linear deformable ones. Thus, when these two modules are integrated, the parameter savings from LDConv outweigh the slight increase from CA, leading to an overall reduction greater than that observed when CA is used in isolation.

Four ablation experiments are carried out to investigate how MGD affects the training process of the LKD-YOLOv8n student network. The results are provided in Table 2.

The results in Table 2 validate the outstanding contribution of MGD in training the LKD-YOLOv8n student model. Group 1 is set to be the baseline model, which is also the origin YOLOv8n model. Group 1 and Group 2 demonstrate the effectiveness of MGD in knowledge distillation, bringing a 0.08% increase in mAP@0.5:0.95 for the student model. A comparison between Group 1 and Group 3 reveals that the integration of the CA mechanism in conjunction with MGD results in a notable performance improvement (2.16%) in mAP@0.5:0.95. Groups 4 refers to the target model under our approach, which simultaneously demonstrates accuracy improvement and model lightweighting. Under the circumstances that the parameter number of the target model decreases by 7.9% and FLOPs is reduced by 7.3%, the mAP50:95 still improves by 1.18%, further highlighting the exceptional performance and effectiveness of our proposed approach. Despite achieving reductions in FLOPs and improvements in mAP@0.5:0.95, our model experiences a drop of approximately 15 FPS, which we attribute to the additional computational overhead introduced by the CA module. We consider this trade-off acceptable given the overall performance.

### 4.5. Comparison with SOTAs

#### 4.5.1. Quantitative Experiments

Additionally, we conduct comparative experiments on several representative object detection frameworks—YOLOv5, YOLOv9, and YOLOv10—to further validate the robustness of our approach. The corresponding results are presented in Table 3.

The quantitative comparison presented in Table 3 indicates that the method we have proposed surpasses the performance of current methodologies on the FLIR Thermal Dataset. Although YOLOv5n has the fewest parameters and FLOPs, its performance is significantly lower than other models, rendering it unsuitable for practical use. YOLOv9t and YOLOv10n have parameters and FLOPs (second least) that are comparable to our model. However, their mAP@50:95 scores are lower by 2.62% and 2.11%, respectively, indicating that their performance is inferior to our model. To further validate the performance of our proposed method, we conducted evaluations using two other well-known YOLO-based models: YOLO-NAS and PP-YOLOE(employing their smallest configurations). Although both models achieved better detection performance compared to our LKD-YOLOv8, their parameter counts and FLOPs are considerably higher. Such computational demands may exceed the capabilities of typical edge devices, limiting their practical deployment in resource-constrained infrared detection scenarios.

To conclude, LKD-YOLOv8 requires fewer parameters and FLOPs while achieving higher detection precision, significantly improving efficiency on low-power computing platforms.

#### 4.5.2. Qualitative Experiments

**Figure 7:**
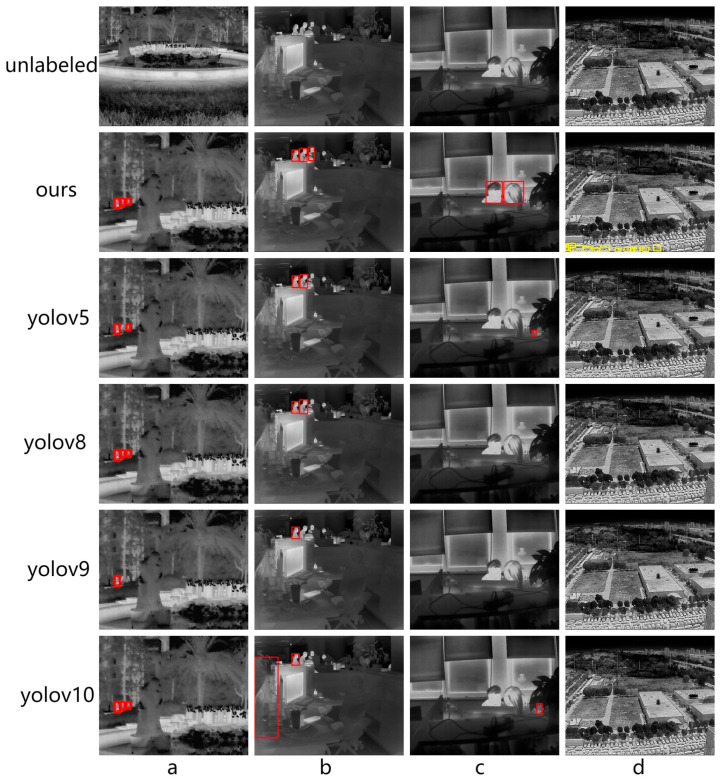
The qualitative results on the testing dataset. To evaluate the performance of the model in different environment, we collected our own testing dataset for evaluation. The red bounding box refers to detected persons, and yellow bounding box refers to detected cars.

The qualitative comparison results are illustrated in Figure 7, which reveal that our model not only achieves a higher recall rate (i.e., detecting target objects), but also maintains a low false positive rate. For instance, in Figure 7a, our model successfully detects all three small-sized persons. In Figure 7b, our model accurately identifies all of the partially occluded persons, a case often missed by other models. Furthermore, in Figure 7c, our model is capable of detecting two individuals with little facial details, whereas other models fail to detect targets with limited texture information. Figure 7d indicates that our model has started to exhibit the ability to detect a cluster of tiny targets that are stacked together, a scenario that other models either fail to detect or misidentify. Although not all vehicles are detected, our model demonstrates notable improvements compared to other models.

### 4.6. Experiments Evaluation Results

**Figure 8:**
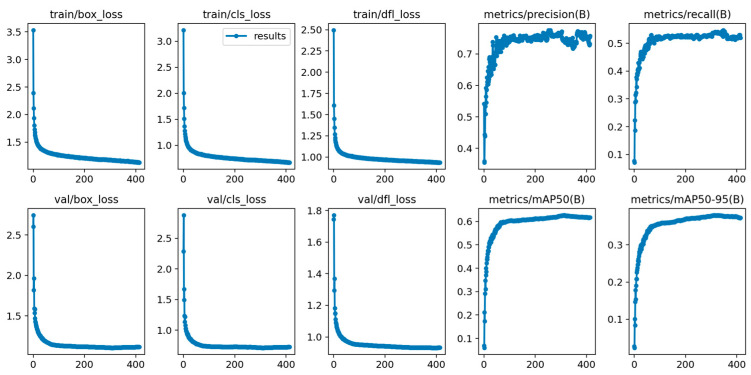
Training and validation metrics of LKD-YOLOv8.

**Figure 9:**
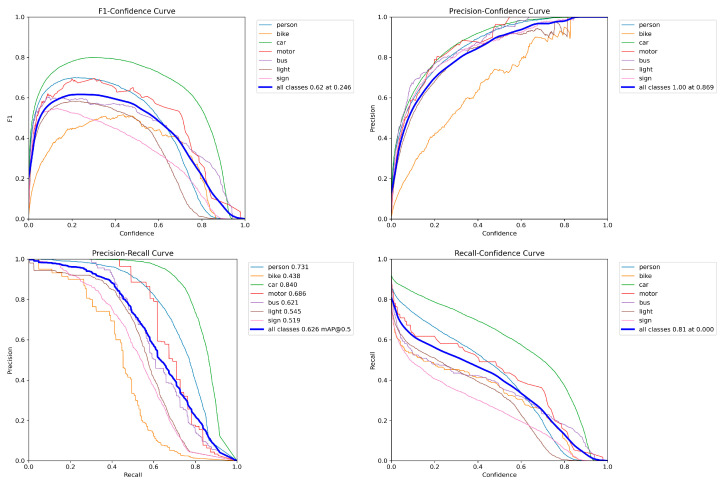
Validation metrics visualization of LKD-YOLOv8.

The training and validation metrics of our LKD-YOLOv8 are shown in Figure 8. The validation metrics visualization of our LKD-YOLOv8 are shown in Figure 9, including the F1–Confidence Curve, Precision–Confidence Curve, Precision–Recall Curve and Recall–Confidence Curve.

### 4.7. Compare with Logit-Based Distillation

In Table 4, we present a comparison of the performance of the logit-based distillation method and the MGD method. The results demonstrate that the MGD outperforms the logit-based method.

## 5. Conclusions

We propose LKD-YOLOv8, a novel lightweight method for infrared object detection that alleviated the issues related to implementing deep learning algorithms on low-power computing platforms. By integrating YOLOv8 with MGD, LDConv, and CA, the proposed model enhances both the accuracy and low-latency inference capability of infrared object detection systems. The combination of knowledge distillation and advanced feature extraction techniques not only improves the feature adaptability, but also ensures efficient performance without a significant loss of accuracy. Experimental results demonstrate that LKD-YOLOv8 outperforms baseline methods with a 1.18% improvement in mAP@0.5:0.95 while achieving a 7.9% reduction in parameter size and a 7.3% reduction in FLOPs. Consequently, the proposed approach maintains a balance between performance and computational expense, demonstrating its potential as a solution for infrared object detection in edge computing contexts, where both performance and resource limitations are critical considerations. Although LKD-YOLOv8 exhibits superior performance, it still has limitations. First, while our model is optimized for inference efficiency, the training process still relies on a relatively high-resource environment due to the use of a large teacher model and multi-stage distillation. Second, due to the extra distillation procedure, the overall training time increases more than threefold compared to standard single-model training, which may pose challenges for scalability and rapid deployment. In future work, we plan to investigate self-distillation techniques or optimize the distillation process to reduce training complexity and enable the faster deployment of models on resource-constrained edge devices.

## Figures and Tables

**Table 1:** Ablation study of modules used in LKD-YOLOv8n model. √ and × indicate whether the module is incorporated or not, respectively.

Group	LDConv	CA	Parameter (million)	FLOPs (G)	FPS	Map@0.5:0.95 (%)
1	×	×	3.01	8.2	196	0.3756
2	√	×	2.65	7.51	217	0.3748
3	×	√	3.02	8.2	192	0.3892
4	√	√	2.78	7.66	202	0.3824

**Table 2:** Ablation study of MGD distilling LKD-YOLOv8n student model. √ and × indicate whether the module or operation is incorporated or not, respectively. Group 4 refers to the target model trained by our proposed approach, which is highlighted for comparative analysis.

Group	Teacher	MGD	LDConv	CA	Parameter (million)	FLOPs (G)	FPS	Map@0.5:0.95 (%)
1	×	×	×	×	3.01	8.2	196	0.3756
2	LKD-YOLOv8s	√	×	×	3.01	8.2	196	0.3889
3	LKD-YOLOv8s	√	×	√	3.01	8.1	175	0.3972
**4**	**LKD-** **YOLOv8s**	**√**	**√**	**√**	**2.77**	**7.6**	**181**	**0.3874**

**Table 3:** Quantitative comparison results with other SOTA models on the Flir Thermal Dataset. Red text indicates the best performance.

Group	Method	Parameter (million)	FLOPs (G)	mAP@0.5:0.95 (%)
1	YOLOv8n	3.01	8.2	0.3756
2	YOLOv5n	1.77	4.2	0.3121
3	YOLOv9t	2.0	7.7	0.3612
4	YOLOv10n	2.69	8.2	0.3663
5	PP-YOLOE-S	7.96	17.36	0.3931
6	YOLO-NAS	12.88	17.52	0.3942
7	LKD-YOLOv8n (ours)	2.77	7.6	**0.3874**

**Table 4:** Comparisions of distillation methods on LKD-YOLOv8. CWD [50] is the channel-wise distillation method.

Group	Method	Map@0.5:0.95 (%)
1	MGD	0.3874
2	CWD	0.3796

## Data Availability

The data that support the findings of this study are available on request from the corresponding author.
